# Supplementary material for: Spectral interferometry with waveform-dependent relativistic high-order harmonics from plasma surfaces
Source: Nat Commun. 2018 Nov 26;9:4992. doi: 10.1038/s41467-018-07421-5 (PMC6255866; doi:10.1038/s41467-018-07421-5)
Supplement: Supplementary file 1 — Supplementary Information [file 41467_2018_7421_MOESM1_ESM.pdf]

Supplementary Information

Spectral interferometry with waveform-dependent relativistic high-order harmonics from plasma surfaces

Dmitrii Kormin[1,2,*], Antonin Borot[1,*], Guangjin Ma[3,4,*], William Dallari[1], Boris Bergues[1,2], Márk Aladi[5], István B. Földes[5], Laszlo Veisz[1,6]

1. Max-Planck-Institut für Quantenoptik, Hans-Kopfermann Strasse 1, 85748, Garching, Germany.
2. Ludwig-Maximilian-Universität München, Am Coulombwall 1, 85748, Garching, Germany.
3. School of Electronics Engineering and Computer Science, Peking University, Beijing 100871, China.
4. Shenzhen SoC Key Laboratory, PKU-HKUST Shenzhen-Hong Kong Institution, Shenzhen 518057, China.
5. Wigner Research Centre for Physics, Hungarian Academy of Sciences, Budapest, Hungary.
6. Department of Physics, Umeå University, SE-901 87 Umeå, Sweden.

* These authors contributed equally to this work.
Correspondence and requests for materials should be addressed to L.V. (email: laszlo.veisz@umu.se)

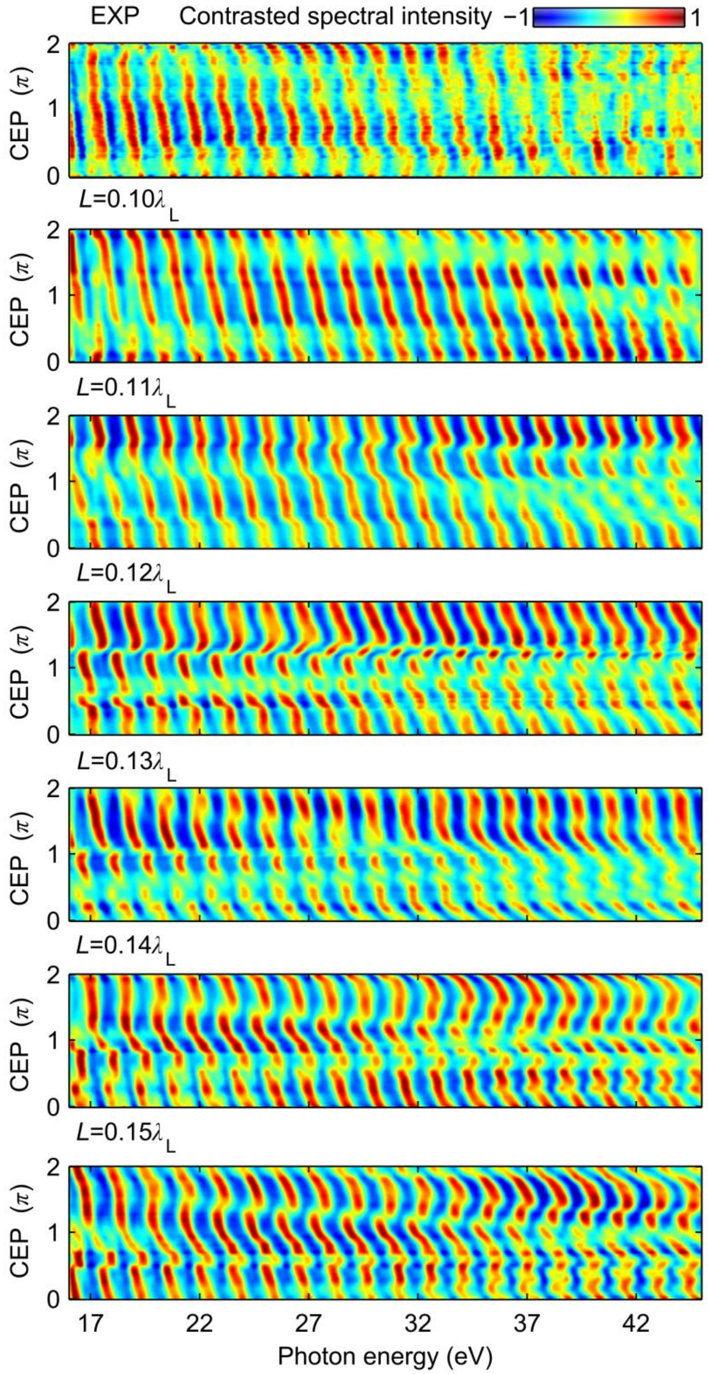

Supplementary Figure 1: CEP and scale length dependence of XUV emission. The measured CEP-sorted XUV spectra and the simulated ones for various scale length values. Additional contrast enhancement procedure (see Methods) was applied to measured and simulated spectra for better visualization. A vertical running average smoothing of the data was applied within the CEP measurement error range of 2×210 mrad. When comparing only these CEP-sorted spectra the $L/\lambda_L = 0.1$ scale length shows the best agreement with experiments.

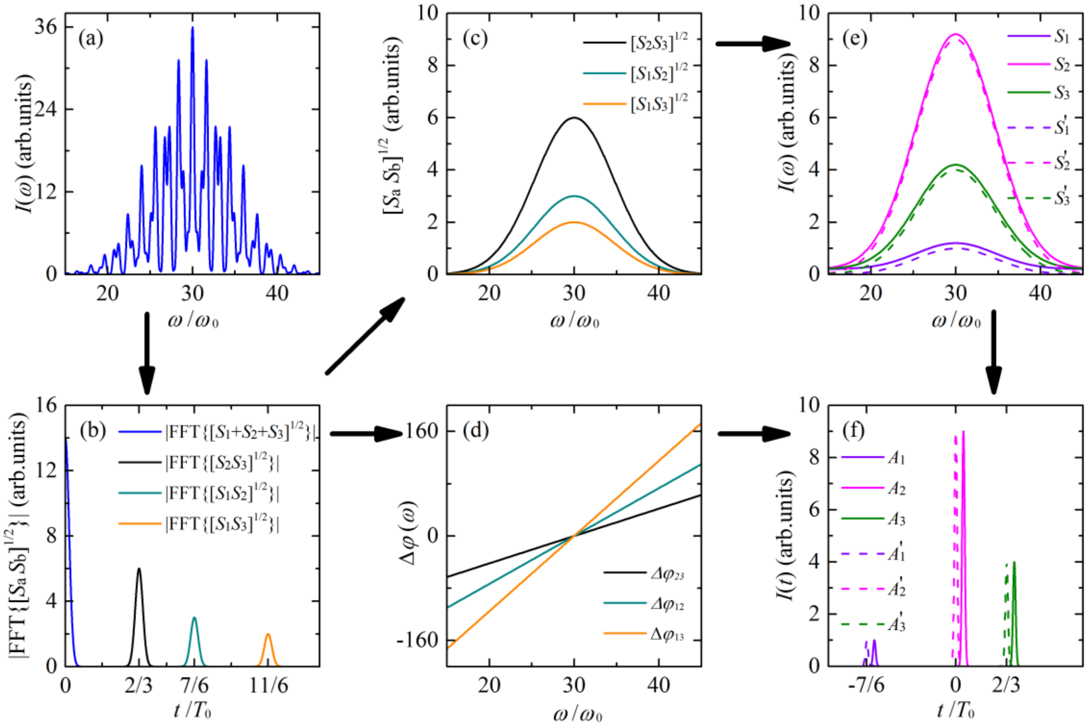

Supplementary Figure 2: Principle of spectral interferometry with three non-equidistant pulses. (a) Modulated spectrum is an interference result of 3 non-equidistant attosecond pulses (APs) with different spectral amplitudes within the same energy range (S_1', S_2', and S_3' in (e)). **(b)** Fourier transform (FT) of this spectrum contains a DC component (blue) and 3 peaks representing an interference of different AP pair combinations. Individual inverse FT of these peaks gives **(c)** the interference spectra $S_{\mathrm{P1}} = \sqrt{S_2 S_3}$, $S_{\mathrm{P2}} = \sqrt{S_1 S_2}$, $S_{\mathrm{P3}} = \sqrt{S_1 S_3}$ as well as **(d)** the phase differences which are related to each other as $\Delta\varphi_{13} = \Delta\varphi_{12} + \Delta\varphi_{23}$. These phase differences include corresponding group delay differences which are also depicted by the FT peaks positions in (b) and fulfill the equation $\Delta t_{13} = \Delta t_{12} + \Delta t_{23}$. **(e)** Individual spectra of APs are reconstructed according to the equations $S_1 = S_{\mathrm{P2}} S_{\mathrm{P3}} / S_{\mathrm{P1}}$, $S_2 = S_{\mathrm{P1}} S_{\mathrm{P2}} / S_{\mathrm{P3}}$, $S_3 = S_{\mathrm{P1}} S_{\mathrm{P3}} / S_{\mathrm{P2}}$ and match the original spectra S_1', S_2', and S_3'. The reconstructed results are vertically shifted by 0.2 for better visibility. **(f)** Temporal structure is obtained by using the spectra and spectral phase differences and assuming no second- or higher-order spectral phase for one of the pulses. It agrees well with original pulse train. The reconstructed results are shifted by 0.1 optical cycle for better visibility. In the above example there is no second- or higher-order spectral phase difference between the three pulses, therefore even without this last assumption the intensity ratio between the pulses remains valid.

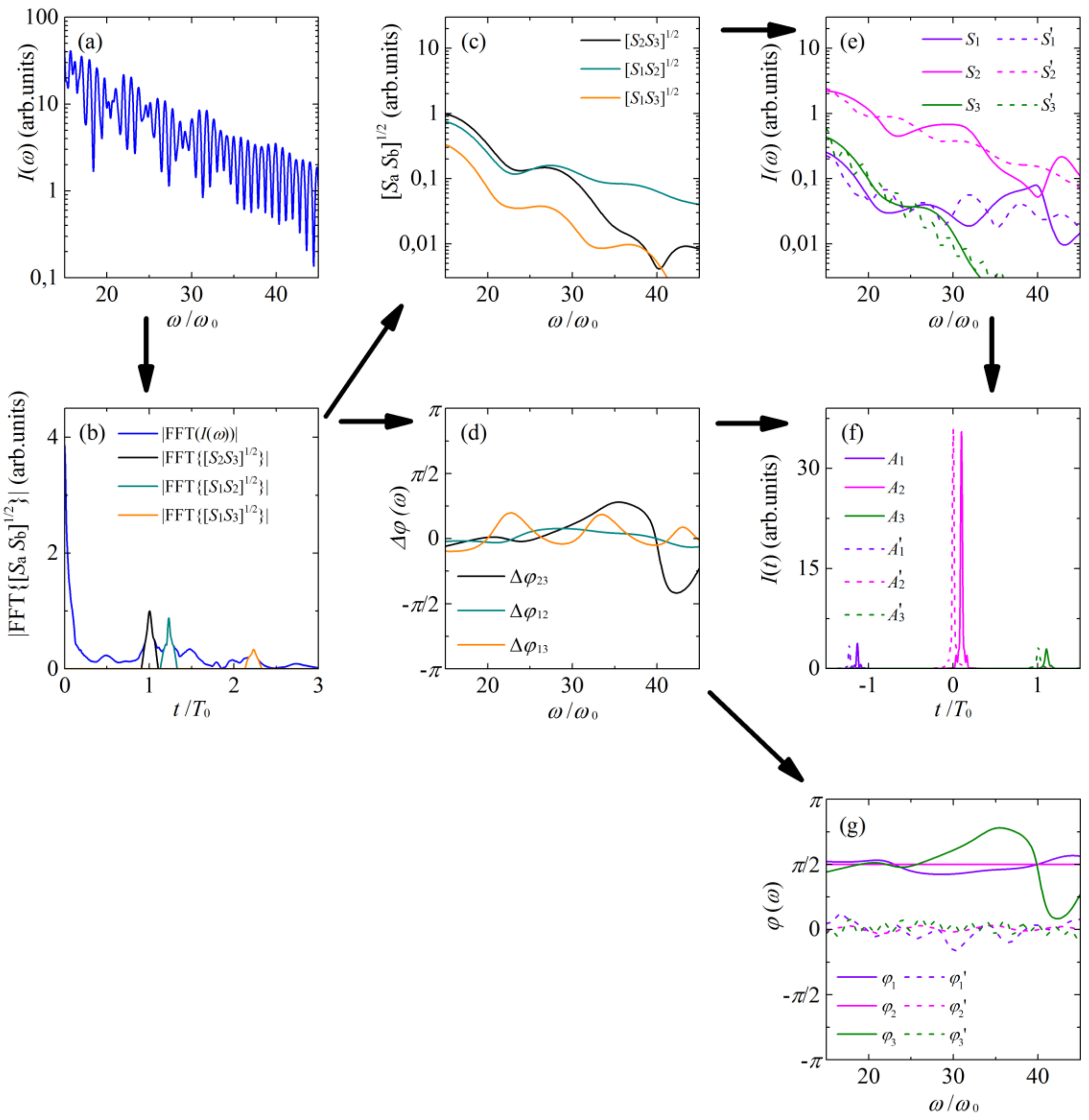

Supplementary Figure 3: Confirmation and application of spectral interferometry with results from PIC simulation. Simulation parameters are $a_0 = 6, L = 0.25\lambda_L, \tau = 5\,\text{fs}, \alpha_{inc} = 45^o, \varphi_{CEP} = 0$. **(a)** Reflected power spectrum is an interference result of 3 APs with different spectral amplitudes within the same energy range (S_1', S_2', and S_3' in (e)). **(b)** FT of this spectrum (blue), containing 3 peaks (black, green, red) representing an interference of different AP pair combinations. Individual inverse FT of these peaks gives **(c)** the interference spectra $S_{P1} = \sqrt{S_2 S_3}$, $S_{P2} = \sqrt{S_1 S_2}$, $S_{P3} = \sqrt{S_1 S_3}$ as well as **(d)** the phase differences $\Delta\varphi_{12}, \Delta\varphi_{23}, \Delta\varphi_{13}$ (linear phase has already been removed). **(e)** Individual spectra of APs are reconstructed according to the equations $S_1 = S_{P2} S_{P3} / S_{P1}$, $S_2 = S_{P1} S_{P2} / S_{P3}$, $S_3 = S_{P1} S_{P3} / S_{P2}$ and match the original spectra S_1', S_2', and S_3'. **(f)** Temporal structure is obtained by using the spectra and spectral phase differences and assuming zero spectral phase for one of the pulses (2nd in this example). It agrees well with original pulse train. The reconstructed results are shifted by 0.1 optical cycle for better visibility. **(g)** Reconstructed spectral phase of the APs $(\varphi_1, \varphi_2, \varphi_3)$ assuming zero value for the second pulse. These phase values are shifted up by $\pi / 2$ for better visibility and reflect well the flat original phase $(\varphi_1', \varphi_2', \varphi_3')$.

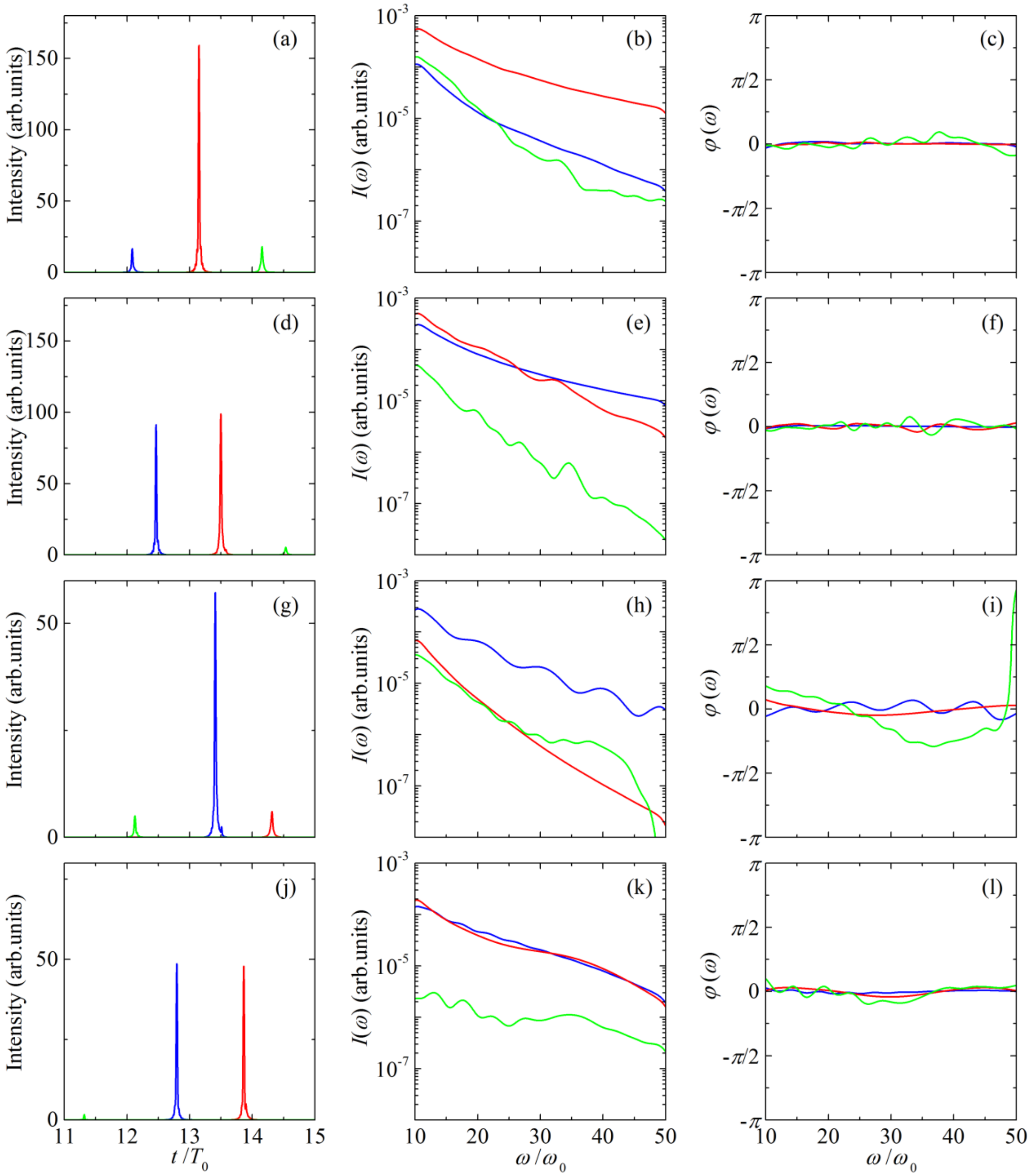

Supplementary Figure 4: Spectrum and spectral phase of individual attosecond pulses from relativistic plasma surfaces. PIC simulations with $a_0 = 6, \tau = 5$ fs parameters. Temporal intensity synthesized from the spectral range H10-H50, spectra of individual APs, and their spectral phase without the linear term for **(a)**, **(b)**, **(c)** $\alpha_{\mathrm{inc}} = 55^{\mathrm{o}}$, $L = 0.1\lambda_{\mathrm{L}}$, φ_{CEP}=2.44 rad , **(d)**, **(e)**, **(f)** $\alpha_{\mathrm{inc}} = 55^{\mathrm{o}}$, $L = 0.1\lambda_{\mathrm{L}}$, φ_{CEP}=0.28 rad , **(g)**, **(h)**, **(i)** $\alpha_{\mathrm{inc}} = 45^{\mathrm{o}}$, $L = 0.4\lambda_{\mathrm{L}}$, φ_{CEP}=4.4 rad , and **(j)**, **(k)**, **(l)** $\alpha_{\mathrm{inc}} = 45^{\mathrm{o}}$, $L = 0.4\lambda_{\mathrm{L}}$, φ_{CEP}=1.47 rad . The obtained spectral phase is flat in all simulated cases, i.e., the APs are Fourier limited.

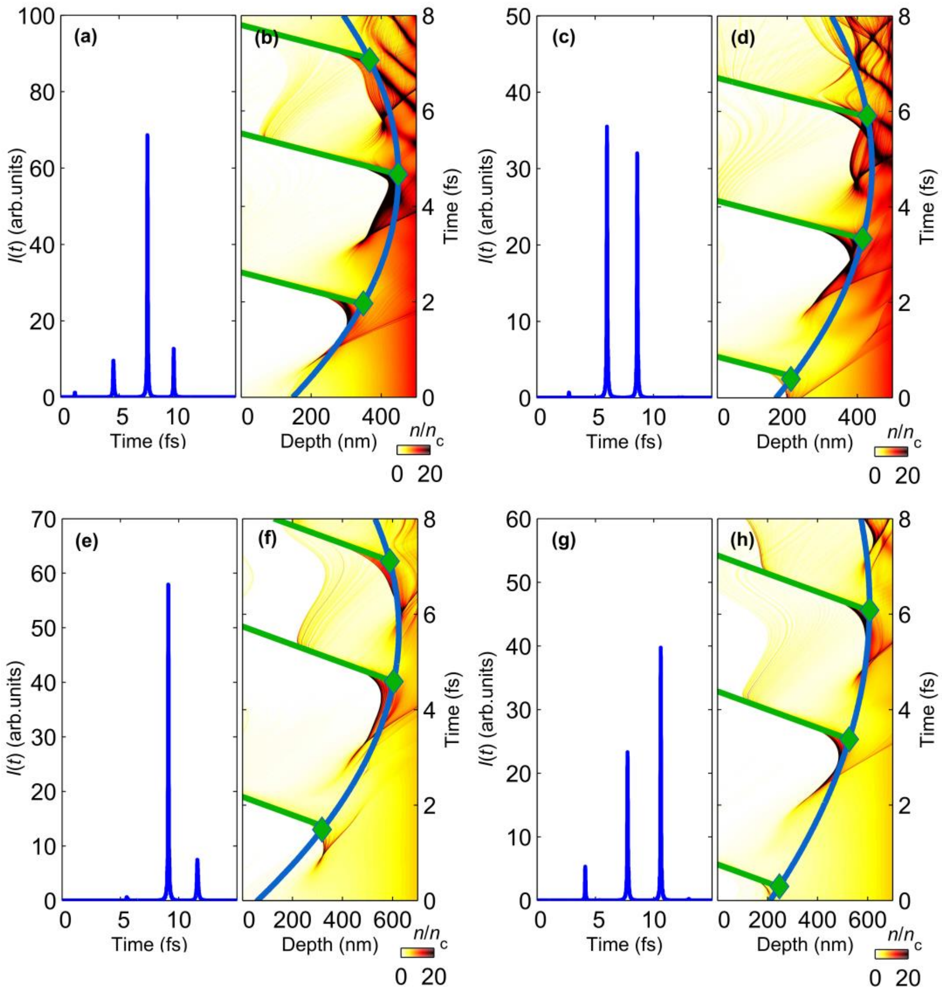

Supplementary Figure 5: Attosecond pulse trains and the plasma surface motion. Temporal intensity and reconstructed parabolas from the corresponding spectrum with the density evolution of the plasma surface for $a_0 = 6, \tau = 5$ fs, $\alpha_{inc} = 55^\circ$ and **(a)**, **(b)** $L = 0.25\lambda_L, \varphi_{CEP} = 0$, **(c)**, **(d)** $L = 0.25\lambda_L, \varphi_{CEP} = \pi$, **(e)**, **(f)** $L = 0.4\lambda_L, \varphi_{CEP} = 2\pi/3$, and **(g)**, **(h)** $L = 0.4\lambda_L, \varphi_{CEP} = 3\pi/2$. The evaluated parabolas reasonably agree with the plasma surface motion in a broad range of scale length and CEP parameters.

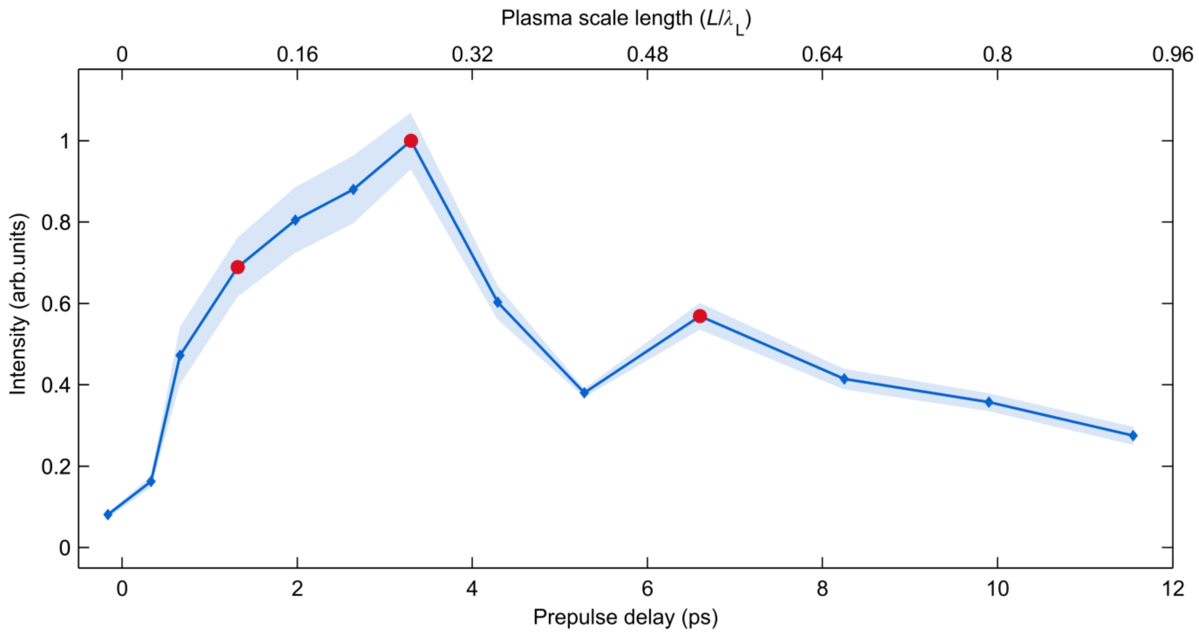

Supplementary Figure 6: Dependence of the measured XUV signal on the prepulse delay. Diamonds represent average XUV signal intensity (integrated over observed spectral range 16–100 eV) for a given prepulse delay, shaded area corresponds to the standard error of the measurement. The upper axis is an approximate scale length from MEDUSA simulations. The shape of the curve is not sensitive for filtering different spectral sub-ranges within the full observation range. Therefore the curve represents dependence of harmonic generation efficiency. The three red points are approximate scale length / prepulse delay positions of the data presented in the manuscript.

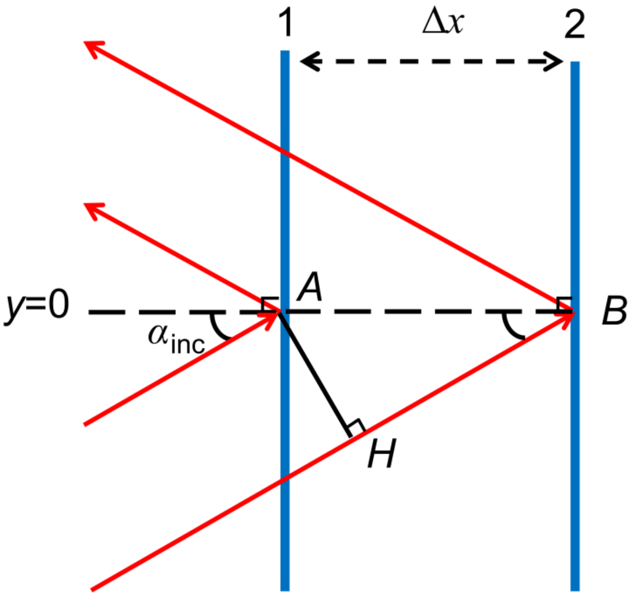

Supplementary Figure 7: Optical path difference in the y=0 plane in the laboratory system. Reflection of the light ray from two planes with Δx shift in normal direction leads to an optical path difference $\Delta d_{opt} = 2HB = 2\Delta x\cos(\alpha_{inc})$ and consequently to different detector arrival time $\Delta t_{arr} = \Delta d_{opt} / c$.

Scale length, L/λ_L	Average exponent, p	Standard deviation, Δp	Average fit quality, R^2
0.13	2.55	0.21	0.95
0.25	2.27	0.17	0.95
0.50	1.87	0.21	0.87

Supplementary Table 1: Power law fit for the HHG spectra. Results of $I_\omega \propto \omega^{-p}$ fit for different scale lengths are listed. The fit parameters are typically averaged over 50-100 spectra including all CEPs for one scale length. Spectra at the longest scale length are influenced more by the beating of 3 pulses and thus the spectrum deviates more from the power law behavior, which results in lower fit quality.
